# Supplementary material for: Quality of Life in Electrochemotherapy for Cutaneous and Mucosal Head and Neck Tumors
Source: J Clin Med. 2021 Sep 24;10(19):4366. doi: 10.3390/jcm10194366 (PMC8509577; doi:10.3390/jcm10194366)
Supplement: Supplementary file 1 [file jcm-10-04366-s001.zip › jcm-1348023-supplementary.pdf]

Supplementary Table S1. Karnofsky Performance status, EORTC QLQ-C30 and H&N35 questionnaires results (means and standard deviations).

Scores	T0	T1 (1 month)	T2 (3 months)	T3 (6 months)
Karnofsky performance status	81.1 ± 16.5	80.0 ± 16.6	83.3 ± 15.7	80.9 ± 14.5
EORTC QLQ-C30 questionnaire				
Global health status	37.6 ± 18.1	46.0 ± 14.5	46.7 ± 14.9	59.8 ± 20.7
Physical functioning	63.2 ± 23.7	64.2 ± 23.5	68.1 ± 19.1	64.2 ± 26.4
Role functioning	57.4 ± 33.4	56.8 ± 28.6	60.2 ± 22.2	63.6 ± 30.6
Emotional functioning	67.9 ± 20.0	74.7 ± 13.4	74.5 ± 11.6	75.0 ± 26.9
Cognitive functioning	71.0 ± 24.3	74.7 ± 27.1	77.8 ± 22.1	84.8 ± 15.7
Social functioning	69.7 ± 24.9	78.4 ± 17.8	76.8 ± 17.3	80.3 ± 28.7
Fatigue	39.5 ± 25.5	43.2 ± 27.4	35.2 ± 23.9	36.4 ± 32.2
Nausea and vomiting	8.0 ± 14.9	9.9 ± 19.7	10.2 ± 21.5	6.1 ± 13.5
Pain	30.9 ± 29.1	23.4 ± 20.3	24.1 ± 21.5	24.2 ± 31.9
Dyspnea	25.9 ± 28.2	19.4 ± 21.2	18.5 ± 17.0	21.2 ± 34.2
Insomnia	32.1 ± 33.9	21.0 ± 22.9	20.4 ± 23.3	24.2 ± 39.7
Appetite loss	29.6 ± 35.0	17.3 ± 26.7	20.4 ± 30.5	24.2 ± 39.7
Constipation	23.4 ± 30.4	14.8 ± 21.3	14.8 ± 20.5	9.1 ± 15.6
Diarrhea	9.9 ± 18.0	2.5 ± 8.9	5.5 ± 12.8	21.2 ± 34.2
Financial difficulties	11.1 ± 22.6	4.9 ± 15.2	5.5 ± 17.1	12.1 ± 30.8
H&N35 questionnaire				
Pain	20.1 ± 17.5	18.8 ± 18.3	18.0 ± 17.4	7.6 ± 12.0
Swallowing	14.8 ± 21.6	20.4 ± 23.3	16.2 ± 19.5	12.9 ± 17.2
Senses problems	18.5 ± 25.0	21.6 ± 24.8	19.4 ± 20.0	13.6 ± 20.8
Speech problems	22.6 ± 20.8	28.4 ± 23.9	24.1 ± 21.6	22.2 ± 22.8
Trouble with social eating	14.8 ± 15.0	21.3 ± 24.2	19.4 ± 19.8	16.7 ± 19.7
Trouble with social contact	12.1 ± 15.8	17.8 ± 17.4	15.9 ± 15.4	15.1 ± 22.7
Less sexuality	28.4 ± 39.7	42.6 ± 37.6	39.8 ± 36.7	40.9 ± 40.4
Teeth	13.6 ± 19.1	12.3 ± 21.0	14.8 ± 23.5	15.1 ± 22.9
Opening mouth	39.5 ± 35.8	28.4 ± 30.2	27.8 ± 34.8	21.2 ± 22.5
Dry mouth	18.5 ± 28.2	13.6 ± 24.9	7.4 ± 18.3	21.2 ± 22.5
Sticky saliva	24.7 ± 23.7	28.4 ± 28.8	24.1 ± 29.8	24.2 ± 26.2
Coughing	14.8 ± 19.2	7.4 ± 16.9	3.7 ± 10.8	3.0 ± 10.0
Felt ill	7.4 ± 16.9	11.1 ± 16.0	14.8 ± 17.0	9.1 ± 21.5
Pain killers	51.8 ± 50.9	51.8 ± 50.9	61.1 ± 50.2	45.4 ± 52.2
Nutritional supplements	33.3 ± 48.0	33.3 ± 48.0	38.9 ± 50.2	9.1 ± 30.1
Feeding tube	3.7 ± 19.2	3.7 ± 19.2	5.5 ± 23.6	9.1 ± 30.1
Weight loss	37.0 ± 49.2	25.9 ± 44.6	27.8 ± 46.1	27.3 ± 46.7
Weight gain	3.7 ± 19.2	11.1 ± 32.0	16.7 ± 38.3	9.1 ± 30.1
